# An integrative systems genetics approach reveals potential causal genes and pathways related to obesity

**DOI:** 10.1186/s13073-015-0229-0

**Published:** 2015-10-20

**Authors:** Lisette J. A. Kogelman, Daria V. Zhernakova, Harm-Jan Westra, Susanna Cirera, Merete Fredholm, Lude Franke, Haja N. Kadarmideen

**Affiliations:** Department of Veterinary Clinical and Animal Sciences, Faculty of Health and Medical Sciences, University of Copenhagen, Grønnegårdsvej 7, 1870 Frederiksberg C, Denmark; Department of Genetics, University of Groningen, University Medical Center Groningen, Groningen, The Netherlands; Divisions of Genetics and Rheumatology, Department of Medicine, Brigham and Women’s Hospital, Harvard Medical School, Boston, MA USA; Partners Center for Personalized Genetic Medicine, Boston, MA USA; Program in Medical and Population Genetics, Broad Institute of MIT and Harvard, Cambridge, MA USA

## Abstract

**Background:**

Obesity is a multi-factorial health problem in which genetic factors play an important role. Limited results have been obtained in single-gene studies using either genomic or transcriptomic data. RNA sequencing technology has shown its potential in gaining accurate knowledge about the transcriptome, and may reveal novel genes affecting complex diseases. Integration of genomic and transcriptomic variation (expression quantitative trait loci [eQTL] mapping) has identified causal variants that affect complex diseases. We integrated transcriptomic data from adipose tissue and genomic data from a porcine model to investigate the mechanisms involved in obesity using a systems genetics approach.

**Methods:**

Using a selective gene expression profiling approach, we selected 36 animals based on a previously created genomic Obesity Index for RNA sequencing of subcutaneous adipose tissue. Differential expression analysis was performed using the Obesity Index as a continuous variable in a linear model. eQTL mapping was then performed to integrate 60 K porcine SNP chip data with the RNA sequencing data. Results were restricted based on genome-wide significant single nucleotide polymorphisms, detected differentially expressed genes, and previously detected co-expressed gene modules. Further data integration was performed by detecting co-expression patterns among eQTLs and integration with protein data.

**Results:**

Differential expression analysis of RNA sequencing data revealed 458 differentially expressed genes. The eQTL mapping resulted in 987 *cis*-eQTLs and 73 *trans*-eQTLs (false discovery rate < 0.05), of which the *cis*-eQTLs were associated with metabolic pathways. We reduced the eQTL search space by focusing on differentially expressed and co-expressed genes and disease-associated single nucleotide polymorphisms to detect obesity-related genes and pathways. Building a co-expression network using eQTLs resulted in the detection of a module strongly associated with lipid pathways. Furthermore, we detected several obesity candidate genes, for example, *ENPP1*, *CTSL*, and *ABHD12B*.

**Conclusions:**

To our knowledge, this is the first study to perform an integrated genomics and transcriptomics (eQTL) study using, and modeling, genomic and subcutaneous adipose tissue RNA sequencing data on obesity in a porcine model. We detected several pathways and potential causal genes for obesity. Further validation and investigation may reveal their exact function and association with obesity.

**Electronic supplementary material:**

The online version of this article (doi:10.1186/s13073-015-0229-0) contains supplementary material, which is available to authorized users.

## Background

Obesity is characterized by an excessive amount of body adipose tissue. Because adipose tissue has many endocrine functions, obesity is a very complex condition and is associated with several severe diseases, such as type 2 diabetes, metabolic syndrome, and several types of cancer. The prevalence of obesity is exponentially rising world-wide [1] and its enormous consequences for the quality of life and life expectancy have led to the need for a better understanding of the molecular pathology involved. To date, genome-wide association studies (GWAS) have identified many different loci associated with obesity and obesity-related phenotypes [2, 3]. However, they explain a limited amount of the 40–70 % predicted genetic variation [2] and provide limited insight into the biological pathways and molecular mechanisms involved.

Transcriptomic analysis can further elucidate the molecular mechanisms, because gene expression provides a link between genetic variations and their corresponding phenotypic alterations [4]. Commonly, transcriptomic data are used to gain biological insight by detecting differences in expression levels [5] between two conditions (i.e., healthy and diseased). Many studies have applied differential expression (DE) analysis to obesity using different tissues, and have detected many biologically relevant genes [6–10]. However, all these studies have used a microarray or quantitative polymerase chain reaction (qPCR) platform. High-throughput RNA sequencing (RNA-Seq) technology has demonstrated advantages beyond microarray technology [11]: the transcriptome is measured more fully and RNA-Seq facilitates the discovery of novel genes. In DE analysis, RNA-Seq data outperform microarray data in terms of the accuracy of measuring gene expression levels and, therefore, RNA-Seq can potentially detect more differentially expressed (DE) genes [12].

Systems genetics approaches via the integration, joint modeling, and analyses of various high-throughput -omics data that represent different levels of biological organization are becoming more popular in genetic studies [13–15]. The integration of genomic and transcriptomic data can be achieved using expression quantitative trait loci (eQTL) studies, whereby genetic variants that underpin differences in expression levels are mapped [14, 16]. It has been shown that eQTLs are highly heritable [17, 18] and have the potential to provide more biological insight into GWAS findings [16]. Several studies have investigated obesity and obesity-related diseases using an eQTL approach, but mainly using microarray expression data [19–21]. Moreover, integration of these data with those from other publically available databases, such as those containing protein–protein interactions (PPI), might also provide further insight into the biological mechanisms behind complex diseases [22, 23].

Here, we present the analysis of RNA-Seq data from subcutaneous adipose tissue from a porcine model specifically created to study obesity. The pig has similar metabolic, physiological, and genetic features to humans, with greater similarity than rodents have to humans, and has shown great potential as a medical model [24, 25]. Extensive discussions on why pigs are a better model are given in our paper describing the pig resource population [26]. We previously showed that the F2 population created for obesity studies demonstrates a high heritability for obesity-related phenotypes [26] and we have detected several obesity-related genes and pathways using network approaches on the genotype [27] and RNA-Seq data [28]. Furthermore, we previously created the Obesity Index (OI), an aggregate additive genetic value for obesity, by combining nine different obesity-related phenotypes [27]. Based on a selective expression profiling design, we selected 36 animals in three groups (lean, intermediate, and obese) for RNA-Seq, and showed that those animals have different metabolic features [28]. In this study, we performed an integrative systems genetics approach to identify causal genes and associated pathways for obesity. This was achieved by integrating information on genetic variants from GWAS (based on high-throughput genotype data) with information from co-expression networks (based on RNA-Seq data) and differential gene expression (based on RNA-Seq data), using an eQTL mapping approach [29, 30]. We also integrated biologically interesting co-expression modules with the known PPI networks to identify key transcriptional and other protein-coding factors underlying the causation of obesity. Thus, the integration of multi-omic biological datasets led to the detection of several obesity-related genes and molecular pathways.

## Methods

### The pig population

The animal resource used in this study was established using breeds that diverged with respect to obesity traits in the parental generation, resulting in a F2 population that was highly divergent with respect to obesity and obesity-related traits. All F2 animals were fed and housed equally. Animal care and maintenance have been conducted according to the Danish “Animal Maintenance Act” (Act 432 dated 09/06/2004) and biological samples were collected according to the Danish “Veterinary Procedures Act” (Act 433 dated 09/06/2004). For a detailed description of the pedigree and the phenotypes, see [26]. In this study, we only used the animals from the Duroc × Göttingen minipig intercross, which has been previously described [28]. In short, 279 F2 animals resulting from an intercross between the Gӧttingen minipig (predisposed for obesity) and Duroc production pig (selected for lean meat content) were intensively phenotyped (e.g., weight, conformation traits, dual-energy X-ray absorptiometry (DXA) scanning, and slaughter characteristics) and genotyped using the Illumina 60 K porcine SNP chip. We previously created and described the OI [27]—a response variable that is an aggregate genotypic value representing the degree of obesity based on the selection index theory [31]. The OI follows a normal distribution; therefore, animals for RNA-Seq of the subcutaneous adipose tissue were selected using a selective gene expression profiling approach [32, 33]. This approach was carefully chosen to ensure sufficient power to detect eQTLs with a relatively small number of animals. In total, we selected 36 animals across three groups: 12 lean, 12 intermediate, and 12 obese animals (Table 1). The family structure (maximizing the number of full siblings to three) and sex distribution was taken into account in the selection of the animals.

**Table 1:** Phenotypic characteristics of selected animals

Group	Number of males	Number of females	Number of unique grandsires	Obesity index	
				Mean	Standard deviation
Lean	7	5	6	−2.47	0.75
Intermediate	6	6	8	0.07	0.11
Obese	6	6	4	2.4	0.36

### RNA sequencing

RNA-Seq was performed as previously described [28]. In short, total RNA was isolated from porcine subcutaneous adipose tissue using the RNeasy Lipid Tissue Mini kit (Qiagen, Hilden, Germany) following the manufacturer’s recommendations. The RNA quantity and quality were assessed by a Nanodrop ND-1000 spectrophotometer and the integrity of the isolated RNA was visually inspected by gel electrophoresis and by measuring the RNA quality indicator value on an Experion system (BioRad, Hemel Heampstead, UK) using the Eukaryote Total RNA StdSens kit (BioRad). Libraries were subsequently constructed using 400 ng total RNA and a TruSeq RNA Sample Prep (Illumina, San Diego, CA, USA) with Poly-A pull down rRNA depletion, following the manufacturer’s recommendations. Samples were sequenced on the HiSeq2500 platform, by dividing the 36 samples over four lanes and using 100 bp paired-end reads. Before alignment, the quality of the reads was checked and the adapters were detected using FastQC. The reads were mapped to the genome assembly SScrofa10.2.72 in STAR aligner using default parameters [34], whereby detected adapters were removed. On average, 20,390 protein-coding genes were detected among the mapped reads. Read counts were estimated using HTSeq [35]. Because transcripts with extremely low expression levels are less reliable [36], transcripts with expression levels equal to or fewer than five counts were removed from the dataset, resulting in 12,253 transcripts per sample. The between-sample bias was removed by estimating the library size factor using the estimateSizeFactor() function in DESeq [37]. Normalization was then performed using the voom() variance-stabilization function in the R-package Limma [38], and samples were corrected for sex and transformed to log_2_-counts per million to approach normality.

### Association of gene expression with degree of obesity

DE genes were detected using a linear model in the R-package Limma [38], taking into account OI as a continuous variable and sex as a factor. First, a linear model was fitted for each gene using the function lmFit() in Limma:

$$ {y}_{ij}={\beta}_{j,OI}O{I}_i+{\beta}_{j, sex}se{x}_i+{\varepsilon}_{ij} $$

where *y*_*ij*_ is the measured expression level of gene *j* for individual *i*, *β*_*j,OI*_ is the estimated regression coefficient from regressing the gene expression value of the *i*^*th*^ individual in its Obesity Index (*OI*_*i*_) for the *j*^*th*^ gene, *β*_*j,sex*_ is the estimated regression coefficient of *sex*, and *ε*_*ij*_ is the error component. Genes were called as DE when the *β*_*j,OI*_was significantly different from 0. Moderated *t*-statistics (ratio of the log2-fold change to its standard error) and log-odds of differential expression by empirical Bayes moderation of the standard errors towards a common value were then calculated using the function eBayes() in Limma. While moderated *t*-test statistics account for differences in the variance of gene expression of a gene across replicates, the log-odds represents the odds of a gene being DE with an OI different from 0. The resulting *P*-values obtained from the moderated *t*-statistic were corrected for multiple testing using the Benjamini-Hochberg procedure [39]. Genes were identified as DE when the adjusted *P*-value false discovery rate [FDR]) was below 0.05. The corresponding gene symbols were reported using BioMart [40].

### eQTL mapping

The integration of the SNP genotype and RNA-Seq data was obtained using an eQTL study approach [13, 29], following the method as described in Westra et al. [30]. The raw expression data obtained by RNA-Seq were quantile-normalized, log_2_-normalized, and centered around zero on a gene level. Finally, a z-transformation was performed on the sample level. Genes were excluded when their expression was below five read counts for any of the samples, because lowly expressed genes potentially introduce a bias. Furthermore, previous studies have shown that the first expression principal components capture experimental variation such as technical and batch effects [41]. Therefore, several eQTL studies were performed on the complete dataset with different principal components removed. In total, four principal components were removed, because this resulted in the highest number of detected eQTLs. The SNPs were included in the genotype data when they had a call rate above 0.95, a minor allele frequency (MAF) above 0.05, and were in Hardy–Weinberg equilibrium (*P* > 1E^−4^). The SNPs were mapped onto the genome using genome assembly SScrofa10.2.74.

Both *cis*- and *trans*-eQTL mapping were performed: eQTLs were considered to be *cis*-acting when the distance between the gene and SNP was less than 1 Mb, and *trans*-acting when the distance was greater than 1 Mb or when the eQTL was located on another chromosome. The relatively large flanking distance of 1 Mb was chosen because haplotype blocks in pigs are larger than in humans and are even larger in F2 populations. Other eQTL pig studies have adapted an even larger flanking distance (e.g., 10 Mb) for assigning *cis*-eQTLs [19, 42]. To correct for multiple testing, we created a null distribution of *P*-values by permuting expression phenotypes relative to genotypes ten times, and then compared the real eQTL *P*-value distribution to the null distribution. eQTLs were only considered to be significant if the FDR was below 0.05. If several eQTLs were detected per gene, the strongest effect for each gene was presented.

The eQTL mapping was first performed on all SNPs and genes that exceeded the quality control thresholds (52,004 SNPs and 12,253 genes). Furthermore, the analyses were restricted to genes that were DE in this study (458 genes) and to SNPs that were significantly associated with the OI, according to our previously published analysis (366 SNPs) [27].

The detected eQTLs were further investigated according to their physical location in the genome and their effects. The distance from the expression SNP (eSNP) to the affected gene was calculated as the distance from the location of the SNP to the location of the transcription start site of the affected gene. The location and the effect of the eSNPs were assigned using the Variant Effect Predictor [43] with the SScrofa10.2.74 assembly, where the results were restricted to the most severe annotation of the SNP variant.

### Integration of eQTLs with co-expression network analysis

We previously conducted a weighted gene co-expression network analysis (WGCNA) on the same RNA-Seq data, and recently published the method and results [28]. To integrate the results from the previous study with those of this study, we evaluated how many eQTLs in the WGCNA modules (clusters of highly interconnected genes) could be detected. We focused solely on the modules that were previously detected as potential biologically interesting: the Blue Module, the Brown Module, the Light-yellow Module, the Black Module, and the Green-yellow Module [28]. Because we are interested in potential causal genes within these modules, we extracted only those genes from the modules that were identified as *cis*-eQTLs.

### Supervised gene co-expression network analysis and the integration of protein–protein interactions

All detected genes in the eQTL mapping and DE analysis were used for supervised WGCNA (sWGCNA). The network was constructed using the framework of Langfelder and Horvath [44], which is also described in Kogelman et al. [27]. Briefly, the adjacency matrix was created by calculating the Pearson’s correlations between the selected genes, and was raised by a power β to reach a scale-free topology index (*R*^*2*^) of at least 0.90. The topological overlap measure (TOM), which assess the degree of shared neighbors between pairs of genes, was calculated based on the adjacency matrix and was used as input for the gene dendrogram (1-TOM). Following this step, modules were detected and assigned to a color as branches of the gene dendrogram using the DynamicTreeCut algorithm [45], using a minimum module size of 25 genes per module. The module eigengene, the first principal component of each module, represents the module’s expression and was used to detect biologically relevant modules. The Module–Trait Relationship (MTR) was calculated as the correlation between the module eigengene and traits of interest, and modules with a significant correlation >0.5 were selected for functional annotation. Genes in the module were retained when their intra-modular connectivity was >0.6, and when their intra-modular connectivity with other modules was >0.6. The module hub gene was detected as the gene in the module with the highest connectivity, or based on a high intra-modular connectivity (>0.8) and high correlation between the individual gene and the trait of interest (>0.6).

Potentially relevant biological modules were visualized in Cytoscape [46] and were integrated with the known PPI from IntAct [47], where corresponding Uniprot accession IDs were extracted using BioMart [40]. The networks of the selected module and PPI were merged and network analysis within Cytoscape was performed to gain insight into the network topology. Subsequently, the community clustering algorithm GLay [48] (in the ClusterMaker app) was used to detect clusters in the merged co-expression and PPI network.

### Functional annotation

Over-represented Gene Ontology (GO) terms and KEGG pathways among the DE genes were detected using the software GoSeq [49], because GoSeq corrects for length bias in RNA-Seq data. First, a probability weighting function for all genes was calculated, based on a given set of biased data for gene length with the function nullp() in GoSeq. Second, a selection-unbiased testing was performed for GO or KEGG enrichment amongst the DE genes. The *P*-values were corrected for multiple testing using the Benjamini-Hochberg method and GO terms and KEGG pathways were detected as being significantly over-represented using a *P*-value of 0.05. The over-represented GO terms and KEGG pathways were separately detected for all DE genes, including the upregulated and downregulated DE genes.

Functional annotation analysis of the detected eQTLs was performed using GeneNetwork (http://www.genenetwork.nl), which detects over-represented GO terms, KEGG pathways, and associated phenotypes and tissues [50]. GeneNetwork is based on expression datasets from humans, mice, and rats, and predicts functions of genes against known pathways in various biological databases. The over-representation of GO terms and pathways was tested using the Mann–Whitney U test, and *P*-values were corrected for multiple testing using the Bonferroni correction.

**Fig. 1:**
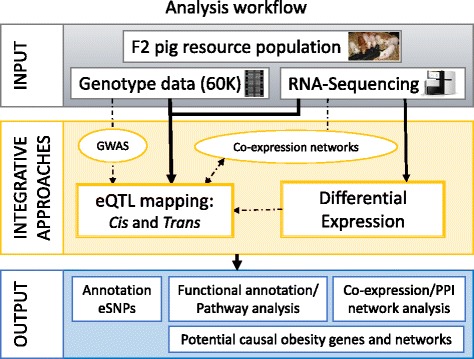
Workflow of the analyses performed in this study. *Circles* represent previously published analyses; *dashed lines* represent the integration of the analysis results. *eQTL* expression quantitative trait loci, *eSNP* expression single nucleotide polymorphism, *PPI* protein–protein interaction

The complete workflow used in this study is presented in Fig. 1.

### Availability of supporting data

The RNA-Seq expression data in this publication have been deposited in NCBI’s Gene Expression Omnibus (GEO) and are available through accession number [GEO:GSE61271].

## Results and discussion

### Association of gene expression with degree of obesity

The detection of DE genes can lead to a better understanding of genetic and biological differences between two different conditions and to the detection of predictive biomarkers [51, 52]. In this study, we have used the level of obesity as a continuous variable, whereby we correct for the effect of sex. In total, we found 458 DE genes (FDR < 0.05), with a *β*_*j,OI*_ ranging from −0.42 to 0.48. All DE results are presented in Additional file 1.

**Fig. 2:**
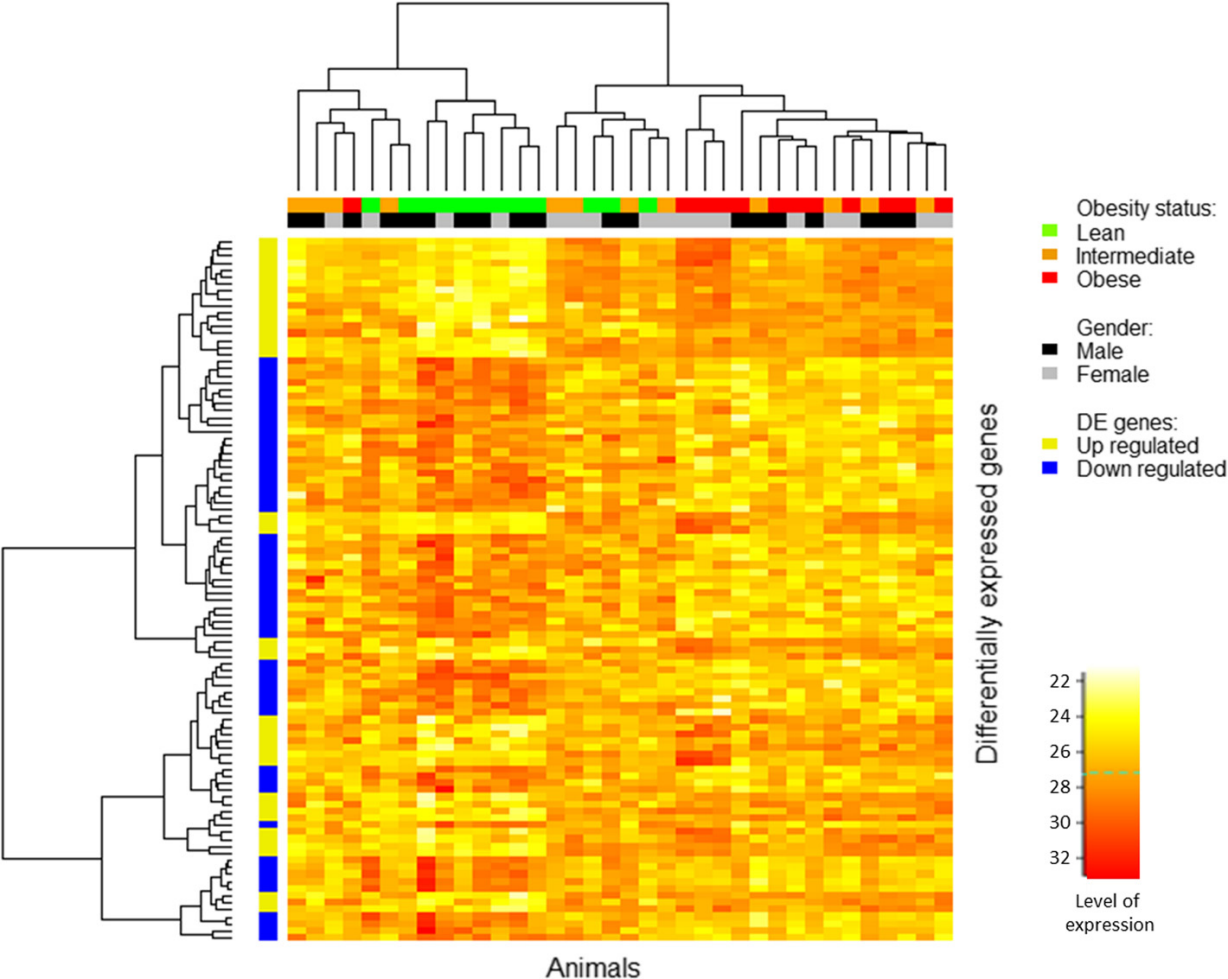
Heatmap of the top 100 differentially expressed genes. The intensity of the yellow/red colors represents the expression level. The *rows* and *columns* have been sorted according to the gene clustering tree. The *left-hand bar* is color-coded according to the upregulation or downregulation in obese animals. The *upper bars* below the animal cluster represents the obesity status and the sex of the animals. *DE* differentially expressed

The heatmap of the top 100 DE genes (Fig. 2) shows that mostly those from the lean and obese animals cluster together within each group. However, those from intermediate animals cluster with both the obese and lean groups. This is possibly due to the OI values of the intermediate group being borderline between the two groups. The genes partly clustered based on the direction of regulation (up or down). The top 10 DE genes include *TAS1R3*, *CSGALNACT1*, *MAML3*, *ROM1*, *LRRC16B*, *EML5*, *RPS12* (all downregulated in obese animals), and *ADAM9* (upregulated in obese animals). We discuss here only selected genes from this group. The *TAS1R3* gene encodes a taste receptor, which has been associated with sweetness responsiveness in mice [53]. Taste receptors influence the perception of food [54], and therefore affect eating behavior [55], which might be associated with obesity [56, 57]. The *CSGALNACT1* (chondroitin sulfate N-acetylgalactosaminyltransferase 1) gene plays a role in the process of glucuronidation: the addition of glucuronic acid to a substrate. This gene has been associated with Bell’s palsy (dysfunctioning of the facial nerve, resulting in facial paralysis) and Morquio Syndrome b (a metabolic disease, characterized by the inability to breakdown glycosaminoglycans). However, this gene also has an important function in the development of osteoarthritis [58], a bone remodeling disease that has been associated with obesity. The 40S ribosomal protein S12 is encoded by *RPS12*, which is associated with diabetic nephropathy in African Americans [59]. The final gene, *ADAM9*, encodes a transmembrane glycoprotein that functions in integrin binding and SH3 domain binding. The *ADAM9* gene is involved in the formation of multinuclear cells and, consequently, with osteoclast fusion [60]; its downregulation might result in reduced bone mass. Bone mineral density is closely related to obesity: it has been suggested that the increased body load on joints resulting from obesity might stimulate the formation of bone mass, even though data from studies remain controversial [61]. However, body adipose tissue is also closely associated with the immune system and bone remodeling (decreased bone mineral density), by the secretion of adipocytes [62]. In a previous study, we identified several genes that might play a role in these associations [28]. The downregulation of these genes in obese animals might correspond to the decreased bone mass via the secretion of adipocytes, but does not correspond to an increased obesity-induced bone mass.

Out of the 458 DE genes, 249 were downregulated and 209 were upregulated in obese animals. Functional annotation analysis was performed for the downregulated and upregulated genes separately. Surprisingly, almost all over-represented pathways were the result of the presence of upregulated genes. The over-represented pathways were mostly associated with the immune system (e.g., Ribosome, *P* = 4.54E^−12^ and leukocyte trans-endothelial migration, *P* = 4.32E^−4^), except for starch and sucrose metabolism (*P* = 6.67E^−4^) and osteoclast differentiation (*P* = 6.67E^−4^). However, osteoclasts are derived from macrophages and therefore are also related to the immune system [63], which was shown previously [28]. Within the GO terms, DE genes showed a striking relationship, mainly among the upregulated genes, with the immune system, (e.g., immune system process, *P* = 2.79E^−20^ and immune response, *P* = 7.92E^−18^). The downregulated genes were mainly involved with functions related to ribosomes and the translational process (e.g., translational termination, *P* = 6.56E^−17^ and cytosolic ribosome, *P* = 1.33E^−15^), which cannot be linked directly to obesity itself.

### The location and annotation of eQTLs

The eQTL mapping led to the detection of 1,070 eQTLs: 987 *cis*-eQTLs and 73 *trans*-eQTLs (FDR < 0.05). All detected eQTLs are presented in Additional file 2. SNPs used for eQTL mapping were filtered based on a high MAF (<0.05). To ensure that this did not affect results owing to the low number of animals, we investigated the MAF of the eSNPs. From those results (not presented), it was evident that most SNPs that were detected as eQTLs had a high MAF. More specifically, only 14 SNPs of the detected eQTLs had a MAF between 0.05 and 0.10. Based on those results and the statistical models used to estimate the eQTL effects, followed by appropriate significance testing, we believe that the results are reliable.

**Fig. 3:**
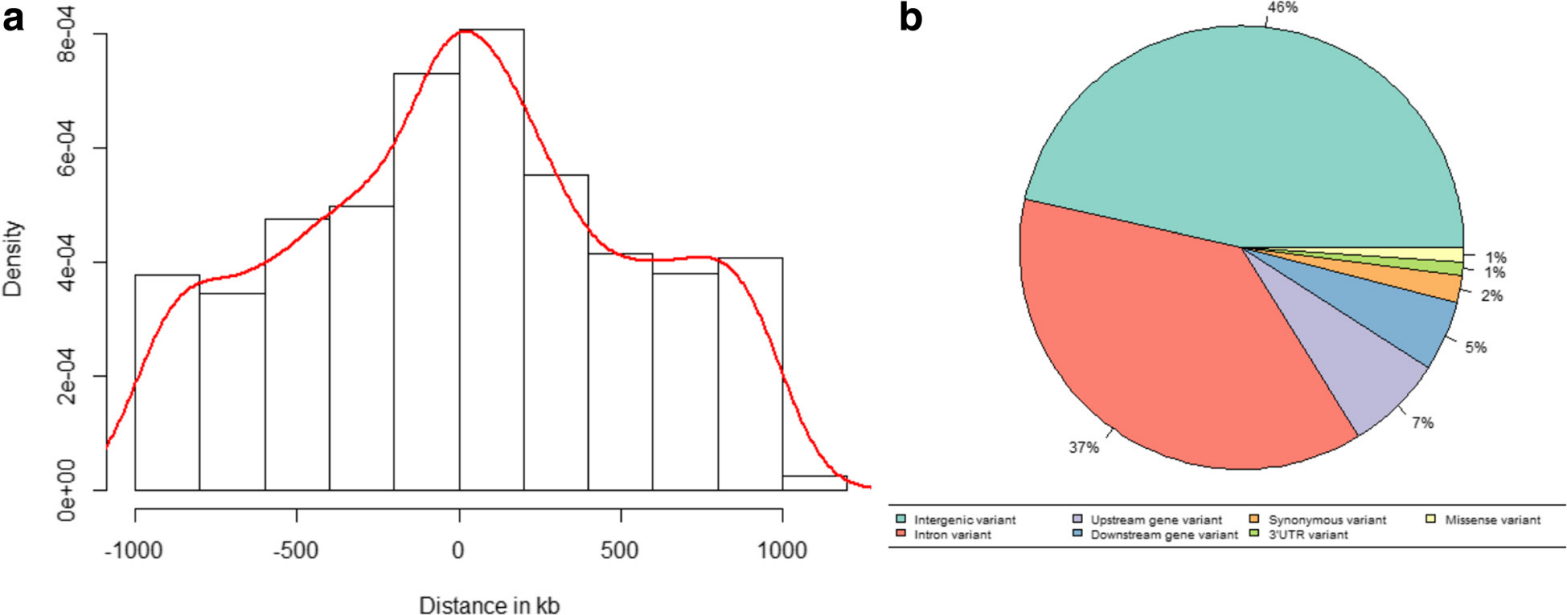
**a** Histogram depicting the distance from the expression single nucleotide polymorphism (eSNP) to the affected transcript of all *cis*-expression quantitative trait loci, with the transcription start site as a base (distance = 0 kb). **b** Visualization of the variant effects of the eSNPs (both *cis* and *trans*) as a percentage. *Kb* kilobase, *UTR* untranslated region

**Fig. 4:**
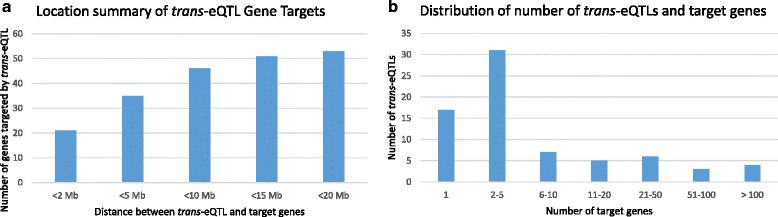
Visualization of the detected *trans*-expression quantitative trait loci (*e-QTLs*). **a** The distance between the single nucleotide polymorphism and target gene of the *trans*-eQTLs. **b** The number of target genes per *trans*-eQTL

The detected eQTLs were annotated as *cis* when the distance between the eSNP and gene was within 1 Mb. This was confirmed by the results, which show that most of the *cis*-eQTLs were located close to the transcription start site, as expected, because this is the region where most transcriptional regulation occurs (Fig. 3a). Further investigation of the effect of the eQTLs (Fig. 3b) showed that most were located in intergenic (46 %) and intronic regions (37 %). In comparison with the complete set of SNPs that passed quality control, we found that the eQTLs were located more often in intronic regions (>8 %) and less often in intergenic regions (<10 %); the frequencies in all other regions were not altered. These percentages agree with the results of previous studies, which showed that disease-associated SNPs are mainly located within non-coding regions (~90 %) [64–66]. We found comparable frequencies of intergenic and intronic SNPs between *cis*-eQTLs and *trans*-eQTLs, where SNPs from *cis*-eQTLs were also located in exonic regions (3′ untranslated region [UTR], 5′UTR, mis-sense, and splice regions). These intergenic and intronic SNPs might be in linkage disequilibrium with a causative SNP, but might also have a regulatory function on the gene expression of the eQTL. Of the detected *trans*-eQTLs, 10 SNPs were located on a different chromosome than the target gene. We further investigated the distance between the SNP and target gene of the *trans*-eQTLs that were located on a similar chromosome, and found that a large part of the *trans*-eQTL would be detected as *cis* by increasing the window size by several megabases (Fig. 4a). Furthermore, we investigated the number of target genes per detected *trans*-eQTL and found that most SNPs were targeting up to five genes (Fig. 4b).

### Functional annotation of eQTLs

Functional annotation analysis using all detected *cis*-eQTLs (987) revealed many metabolism-related GO terms and pathways (Table 2). We also found that several adipose tissues (e.g., adipocytes and subcutaneous adipose tissue) were highly significantly associated with the *cis*-eQTLs.

**Table 2:** GeneNetwork results over all *cis*-eQTLs

	Pathway or process	*P*-value
GO: BP	co-factor biosynthetic process	1 × 10^−18^
	co-factor metabolic process	6 × 10^−18^
	water-soluble vitamin metabolic process	3 × 10^−17^
GO: CC	mitochondrial matrix	5 × 10^−13^
	organelle outer membrane	1 × 10^−12^
	outer membrane	2 × 10^−12^
GO: MF	transferase activity, transferring alkyl or aryl (other than methyl) groups	2 × 10^−17^
	co-enzyme binding	3 × 10^−14^
	co-factor binding	3 × 10^−13^
KEGG	amino sugar and nucleotide sugar metabolism	1 × 10^−12^
	pyrimidine metabolism	4 × 10^−10^
	glutathione metabolism	4 × 10^−9^
Reactome	metabolism of water-soluble vitamins and co-factors	3 × 10^−16^
	metabolism of vitamins and co-factors	3 × 10^−16^
	mitochondrial tRNA amino-acylation	6 × 10^−16^
MGI	abnormal inhibitory postsynaptic currents	3 × 10^−11^
	abnormal cell morphology	7 × 10^−11^
	abnormal circulating amino acid level	9 × 10^−10^
Tissues and cells	adipocytes	1 × 10^−48^
	choroid	3 × 10^−48^
	aortic valve	8 × 10^−47^
	subcutaneous fat	10 × 10^−46^
	subcutaneous fat, abdominal	2 × 10^−45^

Abbreviations: GO Gene Ontology, BP Biological Process, CC Cellular Component, MF Molecular Function, KEGG Kyoto Encyclopedia of Genes and Genomes, MGI Mouse Genome Informatics

In order to identify potential causal genes, we limited the number of eQTL association tests by confining the eQTL mapping to DE genes (458 genes) and, secondly, to obesity-associated SNPs resulting from the previously conducted GWAS on the OI (366 SNPs) [27]. Using the restriction of DE genes, we found a total of 36 eQTLs, among which GO terms and pathways related to cholesterol transport and other lipid process were represented, for example, protein–lipid complex (*P* = 2E^−4^), and lipid digestion, mobilization, and transport (*P* = 2E^−5^). Restriction to GWAS SNPs resulted in the detection of 24 eQTLs. Functional annotation showed that these genes were mainly expressed in adipose tissue (subcutaneous fat, abdominal; *P* = 4E^−3^) and associated phenotypes also showed a link with obesity-related characteristics (e.g., abnormal triglyceride level, *P* = 1E^−3^). Other GO terms and pathways were related to transcription (e.g., viral transcription, *P* = 3E^−4^), metabolism (tyrosine metabolism, *P* = 1E^−3^), or immunity (e.g., influenza infection, P = 2E^−4^). Of those 24 eQTLs, the expression of one target gene was significantly associated (*P* < 0.05) with the OI: *C15orf26*. However, this gene does not seem to have any previously discovered association with obesity or obesity-related diseases. Two other genes tended toward significance: *RAB11A* and *USP36* (*P* < 0.1) *RAB11A* has been shown to be an element in the GLUT4 trafficking machinery [67] and has been associated with glucose metabolism [68]. To our knowledge, *USP36* has not been previously associated with obesity or obesity-related diseases. Unfortunately, we did not identify any overlap of eQTLs between the restricted analyses of the DE subset and GWAS subset.

**Fig. 5:**
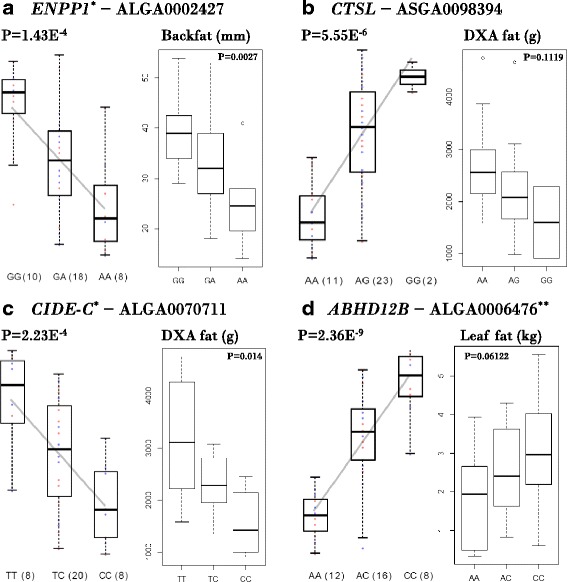
Barplots of the selected expression quantitative trait loci (eQTLs) with one selected phenotype per eQTL (with *P*-value representing the level of significance for difference in phenotype between the two homozygous genotypes). **a**–**c** *cis*-acting eQTLs. **d** *trans*-acting eQTL. * significantly differentially expressed genes, ** significantly associated genome-wide with obesity, *DXA fat* fat estimated using a dual-energy X-ray absorptiometry scan

From the 36 eQTLs detected among the subset of DE genes, 35 were also present in the eQTLs using no restrictions. One of these eQTLs involves the *ENPP1* (ectonucleotide pyrophosphatase/phosphodiesterase 1) gene (Fig. 5a) that encodes a type II transmembrane glycoprotein (*P* = 1.43E^−4^). This glycoprotein plays a role in the regulation of pyrophosphate levels, and several other functions are known: for example, bone mineralization and the regulation of purinergic signaling. More importantly, this gene is highly expressed in adipose tissue and has been associated with obesity, type-2 diabetes, and insulin resistance [69, 70] and also with eating behavior in a pig population [57]. Moreover, expression of this gene inhibits insulin signaling via reduced insulin-receptor tyrosine kinase activity [71]. The data here showed a large difference between the AA and GG phenotypes, whereby the AA animals were lean and GG animals (with an increased expression of *ENPP1*) were obese, which agrees with data in the literature. For example, the AA animals (n = 8) weighed 0.695 kg (standard deviation [SD] = 0.13 kg) at birth and showed a mean gain of 0.37 kg/day (SD = 0.10 kg/day), compared with GG animals (n = 10), which weighed 0.923 kg (SD = 0.12 kg) at birth and gained 0.535 kg/day (SD = 0.12 kg/day). At slaughter, the AA animals contained 24.88 mm of backfat (SD = 7.59 mm) whereas the GG animals had 39.25 mm of backfat (SD = 7.19 mm). However, we found no difference in the fasting glucose levels between the genotypes.

Another eQTL that has been associated with obesity and insulin resistance is *CTSL* (cathepsin L), a lysosomal cysteine proteinase (Fig. 5b, *P* = 5.55E^−6^). Several studies have investigated the role of *CTSL* and have shown, for example, that inhibition of *CTSL* results in limited adipogenesis or lipid accumulation [72] by reducing the levels of pivotal transcriptional mediators of adipogenesis. Moreover, the pharmacological inhibition of *CTSL* resulted in reduced body weight gain, and levels of *CTSL* were elevated in patients who were obese and diabetic [72]. Our results confirm these findings; for example, the AA animals (n = 11) weighed 0.93 kg (SD = 0.16 kg) at birth and showed a mean weight gain of 0.44 kg/day (SD = 0.10 kg/day), whereas the GG animals (n = 2) weighed 0.64 kg (SD = 0.15 kg) at birth and gained 0.32 kg/day (SD = 0.04 kg/day). Furthermore, the AA animals contained 2.73 kg (SD = 0.898 kg) of fat at dual-energy X-ray absorptiometry (DXA) scanning and 2.48 kg (SD = 1.30 kg) of leaf fat at slaughter, whereas the GG animals had 1.60 kg (SD = 0.694 kg) of fat at DXA scanning and 2.20 kg (SD = 1.63) of leaf fat at slaughter. The gene function prediction of GeneNetwork also showed a clear role of *CTSL* in the regulation of cholesterol and lipids, for example, regulation of plasma lipoprotein particle levels (*P* = 2.98E^−16^) and lipid storage (*P* = 3.80E^−14^).

Another detected eQTL was *CIDE-C* (cell death-inducing DFFA-like effector c), also called fat specific protein 27 (*FSP27*), which was more highly expressed in the TT genotype than the CC genotype (Fig. 5c, *P* = 2.23E^−4^). This gene promotes triglyceride (lipid droplet) formation and has a negative regulatory effect on adipocyte apoptosis [73, 74]. A *CIDE-C* knockout model in mice resulted in smaller lipid droplets [75]. Furthermore, *CIDE-C* is regulated by insulin via the Akt1/2-dependent and JNK2-dependent pathways in adipocytes [76]. Animals with the TT genotype (n = 8) were heavier (8.29 versus 17.91 kg at DXA scanning) and showed a higher mean daily gain (0.31 versus 0.51 kg/day) than CC animals (n = 8) and had a considerably higher amount of fat than in CC animals: 1.56 versus 3.20 kg estimated by DXA scanning and 2.01 versus 3.01 kg at slaughter (weight of leaf fat). These results differ from findings in other studies, where *CIDE-C* mRNA levels were lower in obese subjects and were negatively correlated with body mass index and percentage fat mass, but increased in obese patients after weight loss [77]. However, the GEO database also contains studies that show a lower *CIDE-C* expression for high weight gainers versus low weight gainers [GEO: GDS2319] and a higher expression in the adipose stem cells of morbidly obese individuals versus non-obese individuals [GEO: GDS5056]. GeneNetwork identified many adipose-related GO terms and pathways for *CIDE-C* using the predicted function, for example, the GO Biological Process triglyceride metabolic process (*P* = 1.29E^−76^) and GO Cellular Component lipid particle (*P* = 1.27E^−88^).

In addition to the *cis*-eQTLs, we detected 73 *trans*-eQTLs and the functional annotation of this group of genes using GeneNetwork resulted in the detection of (subcutaneous) adipose tissue as associated over-represented tissue (*P* = 6E^−4^). Furthermore, a wide variety of significant GO terms and pathways were detected, which were not all directly linked to obesity, for example, excitatory synapse (*P* = 10E^−5^). In general, *trans-*eQTLs provide a fundamental understanding of potential gene-to-gene regulatory architecture of complex traits and diseases and can also be used to predict transcription factor binding sites [78]. For the *trans-*eQTLs, genes and SNPs were restricted to DE genes and GWAS SNPs. Among the *trans*-eQTLs, only two genes overlapped between all *trans*-eQTLs and those among the DE genes (*GFRα3* and *MYH3*), and only one gene overlapped between all *trans*-eQTLs and the *trans*-eQTLs among the GWAS-significant SNPs (*ABHD12B*). The *GFRα3* gene encodes the artemin receptor, which is a neurotrophin with various functions, such as nerve regeneration and tumor-cell migration [79], although no direct link has previously been found between *GFRα3* and obesity. Similarly, no direct link has been found between *MYH3* (encoding myosin, heavy chain 3, skeletal muscle, embryonic) and obesity. Myosin converts chemical energy into mechanical energy via ATP hydrolysis. Growth characteristics in cattle and pigs have been associated with *MYH3* expression, in addition to a difference in muscle growth between and lean and obese pigs, which suggests an association between *MYH3* and adiposity. However, we observed no difference in obesity-related phenotypes according to *MYH3* expression. The third gene, *ABHD12B* (α/β-hydrolase domain containing 12B), has also not been previously associated with obesity (Fig. 5d, *P* = 2.36E^−9^). However, it plays a role in lysophosphatidylserine (LPS) metabolism, and *ABHD12B* knockout mice have a deregulated accumulation of proinflammatory lipids [80]. Furthermore, it has been shown that LPS stimulates glucose transport in adipocytes [81]. In this study, animals with the AA genotype on ALGA0006476 (n = 12) were more obese than animals with the CC genotype (n = 8) and showed a mean daily gain of 0.47 kg/day (SD = 0.11 kg/day) compared to 0.38 kg/day (SD = 0.12 kg/day), and a weight of leaf fat at slaughter of 3.06 kg (SD = 1.36 kg) compared to 1.81 kg (SD = 1.23 kg). The expression of *ABHD12B* was higher in CC, suggesting that upregulation or activation of this gene results in leaner animals. To our knowledge, no other studies have shown significant effects for the expression of *ABHD12B*.

### Integration of eQTL results with gene co-expression network analysis

Previously, we investigated the RNA-Seq data from this study using a gene co-expression network approach (WGCNA) [28] and we detected five modules that were potentially biologically associated with obesity-related characteristics. We hypothesized that modules containing more eQTL genes than expected by chance would pinpoint modules that are more likely to be causal for the trait under study. Therefore, we investigated how many eQTLs (out of the 987 detected *cis*-eQTLs) were present in each of the five modules detected using WGCNA. We found five eQTLs in the Green-yellow Module (47 genes), five eQTLs in the Brown Module (86 genes), two eQTLs in the Blue Module (69 genes), and one eQTL in the Black Module (36 genes), with no eQTLs in the Light-yellow Module. This represents 10.64, 5.81, 2.90, 2.78, and 0 % of the number of genes in that particular module, respectively, which is unfortunately not higher than expected by chance (hypergeometric test). The eQTL in the Black Module is a novel gene (uncharacterized protein), with no known orthologs.

The Green-yellow Module was strongly associated with obesity-related phenotypes, but in our previous study, the functional annotation did not identify a relationship with obesity. We have now found five eQTLs in this module: *ALDH1L2*, *GGTA1*, *KRR1*, *ME3*, and *OPTN*. The *OPTN* gene encodes optineurin, a protein that has been investigated intensively in the neuroscience field, and is associated with primary open-angle glaucoma and amyotrophic lateral sclerosis [82]. Notably, it also plays a role in adipogenesis, and modulates the developmental switch into brown preadipocytes [83]. GeneNetwork predicts the *adipocytokine signaling pathway* (*P* = 1.5E^−7^) as the most likely associated KEGG pathway. For the other genes, no obvious association with obesity or obesity-related phenotypes was found.

In the Brown Module, we found five eQTLs, representing four unique genes: *ARF6*, *PMVK*, *MSRB2*, and two eQTLs in *RIN2.* The *ARF6* gene encodes a small GTP-binding protein that regulates vesicular trafficking actin cytoskeletal dynamics [84]. The *PMVK* gene encodes an enzyme that functions in the cholesterol biosynthesis pathway, which converts mevalonic acid-5P to mevalonic acid 5-pyrophosphate. Furthermore, it has been shown to be critical in the regulation of the secretion of insulin in pancreatic β cells [85]. The other genes have not been related to obesity or obesity-related phenotypes.

In our previous study, the Blue Module revealed a potential genetic association between obesity, the immune system, and bone remodeling (osteoporosis). Therefore, we would expect that the eQTLs in this module (*LAT2* and *IGSF6*) have a more causal role in this genetic association. Both genes play a role in the immune system, but have not been previously shown to be directly related to obesity.

### Supervised gene co-expression network and integration with protein–protein interactions

**Fig. 6:**
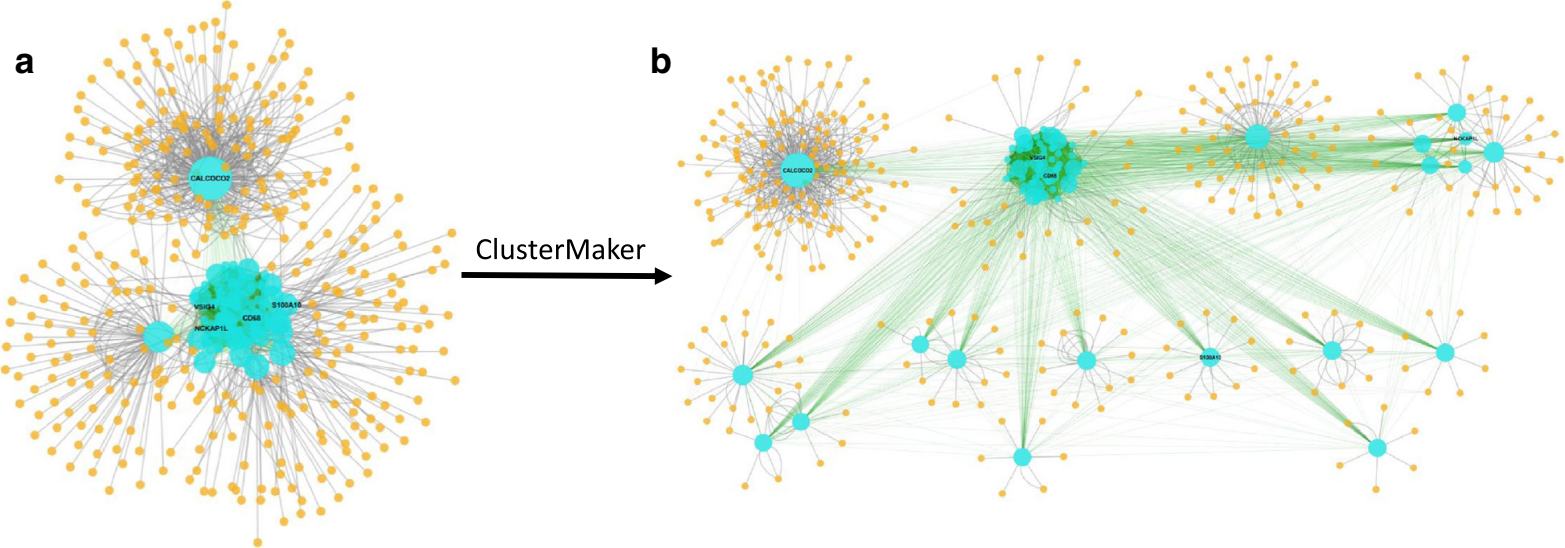
Visualization of the *Turquoise*_sWGCNA_ Module in Cytoscape. **a** The complete network and (**b**) clustering of the network based on GLay community clustering algorithm. Genes in the Turquoise Module are *turquoise* and interacting proteins are *orange*. The size of the nodes is dependent on the betweenness centrality of the node. Edges are *green* to represent a gene–gene interaction (the darkness depends on the weight of the correlation) and *gray* represents protein–protein and gene–protein interactions

Both DE genes and *cis*-eQTL genes were used as input for sWGCNA, with the focus on potential causal genes for obesity. The Pearson’s correlations among 1,408 unique genes (987 *cis-*eQTLs and 458 DE genes) were calculated and raised to a power β, of three, reaching a scale-free topology index (*R*^*2*^) of 0.93. Using the TOM, we detected eight modules of at least 25 genes per module, three of which showed strong significant correlations with the OI (MTR_OI_) and other obesity-related traits: the *Yellow*_sWGCNA_ Module (MTR_OI_ = −0.74), *Blue*_sWGCNA_ Module (MTR_OI_ = −0.71), and *Turquoise*_sWGCNA_ Module (MTR_OI_ = 0.69). The functional annotation of those modules showed strongly significant GO terms and pathways for only the *Turquoise*_sWGCNA_ Module, which were related to obesity and the biological processes lipid localization (*P*_adj_ = 5.35E^−12^) and lipid transport (*P*_adj_ = 2.39E^−10^) were most significantly over-represented. Based solely on the connectivity of the genes in this module, *ITGB2* (β2-Integrin) is the hub gene (highest connectivity) of this module; however, it is not included in the module after gene selection based on the gene-trait correlation (correlation of 0.59 with the OI). According to the gene network prediction of GeneNetwork, *ITGB2* is co-expressed with many other genes, which all possess functions within the immune system (e.g., activation of immune response). This gene has also previously been associated with obesity: mutations in *ITGB2* have been associated with obesity in mice [86], and fat oxidation and insulin metabolism were impaired in knockout mice [87]. The *ITGB2* gene has also been associated with obesity in humans: a polymorphism in this gene is associated with obesity among Japanese individuals living in the US (westernized diet) [88]. Based on measures other than connectivity, such as intra-modular connectivity and gene-trait correlation, other important genes were detected within this module: *NCKAP1L*, *S100A10*, *VSIG4*, and *CD68*, which have all been associated with immune-related processes. The *NCKAP1L* gene is also strongly co-expressed with many other genes but is not expressed in adipose tissue, and functions in immune-related processes. *S100A10* encodes the protein p11, which functions in cellular processes such as exocytosis and endocytosis, and has been associated with serotonergic signaling and, consequently, with depression [89]. *VSIG4* encodes a B7 family-related protein and negatively regulates T cell activation and IL-2 production [90]. *CD68* encodes a glycoprotein that is mainly expressed by monocytes and tissue macrophages [91] and binds to oxidized low-density lipoproteins on the cell surface, and might consequently play a role in atherosclerotic lesions [92]. We further analyzed and visualized this module in Cytoscape and merged the turquoise network with the known PPIs from IntAct. The resulting network consisted of 419 nodes and 3,015 edges (Fig. 6a). We calculated the network statistics and clustered the genes and proteins within Cytoscape (Fig. 6b). The largest cluster contained 137 proteins that interacted with a single gene: *CALCOCO2* (calcium binding and coiled–coil domain 2). This gene is significantly upregulated in adipose tissue (*P* = 0.0003) according to HumanMine (http://humanmine.org) and has been shown to be negatively correlated with the level of triglycerides in muscle tissue in a mouse model for human obesity [93]. Two out of the four genes that were detected as potentially important or hub genes (*VSIG4* and *CD68*) were located within second largest module. The third gene (*NCKAP1L*) formed a cluster together with five other genes. The fourth gene (*S100A10*) formed a cluster by itself, with ten other proteins.

## Conclusion

In this study, we examined the transcriptome and genome of 36 lean, intermediate, and obese pigs using a variety of multi-omic systems genetics approaches, with the aim of detecting potential causal genes and regulatory networks for human obesity, using the pig as a model. We performed DE analysis, weighted gene co-expression network analyses, and integrative systems genetics analyses of obesity by integrating and jointly analyzing the genome and transcriptome using an eQTL approach. We also generated networks using the identified eQTLs to provide causal networks and to identify more biologically relevant causal genes.

We successfully identified many DE genes and the associated pathways showed several immune-related pathways and GO terms, mainly among the upregulated genes. Furthermore, we conducted eQTL mapping, a systems genetics approach, to detect which genetic variants affect the expression levels of obesity-related genes. We detected many *cis*-acting and *trans*-acting eQTLs, mostly located in intronic and intragenic regions, which were further analyzed by pathway analyses and we detected many different metabolic pathways using GeneNetwork. To limit our eQTL search to the most promising and potential causal genes, we focused on DE genes and SNPs that exceeded the genome-wide significance threshold in a GWAS with the OI scores. We evaluated how many of the detected eQTLs were present in clusters of highly interconnected genes detected in our previous study, because those clusters containing many eQTLs might represent a causal function of the module leading to obesity. The restriction of the data in these ways led to a subset of eQTLs that were further analyzed using GO and pathway analysis, which resulted in several adiposity-related terms and pathways. The detected *trans*-eQTLs could not be directly linked to obesity, but provided insights into complex *trans*-regulatory mechanisms. Finally, we performed a sWGCNA on all detected *cis*-eQTLs and identified several modules that highly correlated with obesity phenotypes. One of these modules showed a strong association with lipid pathways. However, integration with known PPIs from a publically available database did not provide further insight into important underlying mechanisms.

For years, DNA markers have been studied in association with complex traits, for example, obesity, which has led to the detection of several associated genes. Similarly, gene expression has been studied in detail to detect associated genes. However, the combination of DNA markers and gene expression data leads to a better understanding of the mechanisms behind the translation from DNA marker via transcription toward complex disease, and therefore targets a greater number of potentially causal genes. In this study, we detected several eQTLs that revealed genes that may cause obesity, due to the combined association of DNA marker and transcription with obesity. These genes have been previously associated with obesity-related traits, but have not all been associated with obesity. In this study, we identified, for example, the genes *ENPP1*, *CTSL*, *CIDE-C*, and *ABHD12B* as potential causal genes for obesity, and further validation (e.g., by qPCR in a large human population) and investigation of these genes might lead to biomarkers for obesity. However, in our study we selected the strongest eQTL effect per DNA marker/gene target, but owing to linkage disequilibrium the identified gene might not always be the true causal gene. Therefore, other strategies are needed to prove causality of our detected potentially causal genes, for which several integrative approaches are available, for example, as proposed by Schadt et al. [94].

In conclusion, this systems genetics study (integrating RNA-Sequencing transcriptomic and genomic information) revealed potential causal genes, and provided insight in the genetic and regulatory architecture of obesity pathways. Furthermore, several relevant GO terms and molecular pathways related to obesity, are presented here. To the best of our knowledge, this is the first study to report integrated transcriptomic and genomics data in a porcine model for obesity.

## Additional files

Additional file 1:
**Differentially expressed genes between the different obesity levels.** (XLSX 59 kb) Additional file 2:
**Detected** ***cis*** **-eQTLs and** ***trans*** **-eQTLs in the complete dataset.** (XLSX 90 kb)
